# In-hospital mortality is associated with inflammatory response in NAFLD patients admitted for COVID-19

**DOI:** 10.1371/journal.pone.0240400

**Published:** 2020-10-08

**Authors:** Roberta Forlano, Benjamin H. Mullish, Sujit K. Mukherjee, Rooshi Nathwani, Cristopher Harlow, Peter Crook, Rebekah Judge, Anet Soubieres, Paul Middleton, Anna Daunt, Pablo Perez-Guzman, Nowlan Selvapatt, Maud Lemoine, Ameet Dhar, Mark R. Thursz, Shevanthi Nayagam, Pinelopi Manousou

**Affiliations:** 1 Liver Unit/Division of Digestive Diseases, Department of Metabolism, Digestion and Reproduction, Faculty of Medicine, Imperial College London, London, United Kingdom; 2 MRC Centre for Global Infectious Disease Analysis, Department of Infectious Disease Epidemiology, School of Public Health, Imperial College London, London, United Kingdom; Kaohsiung Medical University, TAIWAN

## Abstract

**Background & aims:**

Although metabolic risk factors are associated with more severe COVID-19, there is little evidence on outcomes in patients with non-alcoholic fatty liver disease (NAFLD). We here describe the clinical characteristics and outcomes of NAFLD patients in a cohort hospitalised for COVID-19.

**Methods:**

This study included all consecutive patients admitted for COVID-19 between February and April 2020 at Imperial College Healthcare NHS Trust, with either imaging of the liver available dated within one year from the admission or a known diagnosis of NAFLD. Clinical data and early weaning score (EWS) were recorded. NAFLD diagnosis was based on imaging or past medical history and patients were stratified for Fibrosis-4 (FIB-4) index. Clinical endpoints were admission to intensive care unit (ICU)and in-hospital mortality.

**Results:**

561 patients were admitted. Overall, 193 patients were included in the study. Fifty nine patients (30%) died, 9 (5%) were still in hospital, and 125 (65%) were discharged. The NAFLD cohort (n = 61) was significantly younger (60 vs 70.5 years, p = 0.046) at presentation compared to the non-NAFLD (n = 132). NAFLD diagnosis was not associated with adverse outcomes. However, the NAFLD group had higher C reactive protein (CRP) (107 vs 91.2 mg/L, p = 0.05) compared to non-NAFLD(n = 132). Among NAFLD patients, male gender (p = 0.01), ferritin (p = 0.003) and EWS (p = 0.047) were associated with in-hospital mortality, while the presence of intermediate/high risk FIB-4 or liver cirrhosis was not.

**Conclusion:**

The presence of NAFLD *per se* was not associated with worse outcomes in patients hospitalised for COVID-19. Though NAFLD patients were younger on admission, disease stage was not associated with clinical outcomes. Yet, mortality was associated with gender and a pronounced inflammatory response in the NAFLD group.

## 1. Introduction

COVID-19 is an infectious disease caused by severe acute respiratory syndrome coronavirus -2 (SARS-CoV-2), a newly discovered member of the coronavirus family which includes also the severe acute respiratory syndrome coronavirus (SARS-CoV) and the Middle East respiratory syndrome coronavirus (MERS-CoV) [1]. The SARS-CoV-2 causes predominant respiratory disease and may lead to acute respiratory distress syndrome (ARDS), but may also affect other organ systems and cause intestinal, hepatic and neuronal disease, with multiple organ failure (MOF), and death, potentially occurring in severe cases [2, 3]. Interestingly, increasing evidence suggest that a cytokine release syndrome-like (CRS) is responsible for adverse outcomes in the subset of critically ill patients [4]. By March 2020, the number of cases and countries affected increased dramatically to the extent that the World Health Organisation characterised the COVID-19 situation as a pandemic [5].

According to preliminary results, an older age and/or the presence of pre-existing metabolic conditions such as diabetes, hypertension and cardiovascular disease define a category of patients at high risk for severe COVID-19 [6]. Interestingly, these risk factors also identify a group of patients at higher risk for Non-Alcoholic Fatty Liver Disease (NAFLD) [7]. NAFLD encompasses a wide spectrum of liver disease, from simple steatosis to Non-Alcoholic steatohepatitis (NASH), with a variable degree of fibrosis to cirrhosis [7]. Overall, NAFLD is considered the leading cause of chronic liver disease worldwide with an estimated global prevalence of 25.4%, which becomes even higher among those with metabolic risk factors [8]. However, as there is currently no established screening, a large number of cases with significant liver disease remain undetected [9].

Patients with NAFLD might be particularly susceptible to severe disease from COVID-19 as they carry a particular combination of risk factors. Firstly, the presence of type-2 diabetes, which is highly prevalent in this group, confers an additional susceptibility to infection *per se*, regardless of long-term glycaemic control [10]. Secondly, considering a higher prevalence of cardiovascular disease, patients with NAFLD may show a decreased cardiac reserve and impaired response in a critical care setting [11, 12]. Finally, given the high rates of obesity, patients with NAFLD may frequently present with co-existing chronic lung disease (i.e. obstructive sleep apnoea syndrome, restrictive lung disease), characterised by difficult intubation and reduced response to ventilation [13]. Nevertheless, at present, there is still little knowledge on how the NAFLD population is affected by the COVID-19 pandemic [14, 15].

In this study, we aimed to describe the clinical characteristics and outcomes of patients with NAFLD admitted and diagnosed with COVID-19 compared with non NAFLD COVID-19 positive admissions. Furthermore, we explored the association between risk factors and clinical outcomes in this group.

## 2. Materials and methods

### 2.1 Study population

This retrospective study included all consecutive adult patients admitted and diagnosed with COVID-19 at Imperial College Healthcare NHS Trust (London, United Kingdom) between the 25^th^ February 2020 and the 5^th^ April 2020. Only patients who had imaging of the liver (either ultrasound or computerised tomography) dated within 1 year from the admission for COVID-19 or a known diagnosis of NAFLD were included in the analysis.

The NAFLD group was compared with all patients with available imaging within one year before the admission. At the time of presentation, a full range of clinical, demographic and laboratory parameters were recorded. As part of the COVID-19 risk-stratification pro-forma set up in our Trust, all patients were specifically interrogated for alcohol consumption, drug history and medications. NAFLD was diagnosed based on imaging or past medical history, while patients with excessive alcohol consumption and other causes of liver disease [16] were excluded. For those with NAFLD, Fibrosis-4 index (FIB-4) was calculated based on the published formula [17] derived from blood tests dated within 1 year before the admission for COVID-19.

As per standard of care, SARS-CoV-2 was detected in naso-pharyngeal swabs using a real-time reverse transcriptase-polymerase chain reaction (RT-PCR) method. Chest radiographs or CT scans were also performed according to clinicians’ evaluation and were interpreted in line with hospital and professional guidelines [18]. Heart rate (HR), respiratory rate (RR), blood pressure (BP) and temperature (TC) were recorded at the time of the hospital admission. The early warning score (EWS) was also calculated based upon physical observations and neurological status at the time of the admission [19]. Details regarding the time interval between the onset of symptoms and hospital admission as well as clinical evolution (such as oxygen requirement, invasive and non-invasive ventilation rates) were recorded. Comorbidities were included as per patients’ medical history. Clinical outcomes were defined as rates of admission to intensive care unit (ICU) and in-hospital mortality and were monitored until the 10^th^ June 2020.

As per hospital guidelines, patients were stratified according to the CRS grading system which is based on the presence of fever, levels of C reactive protein (CRP) and oxygen requirements at presentation [20]. In our Trust, the CRS grading system is used to identify patients at higher risk of clinical deterioration.

### 2.2 Statistical analysis

The distribution of variables was explored using the Shapiro-Wilk test. Since the data were non-normally distributed, continuous variables were expressed as medians and interquartile range (IQR), while categorical variables were expressed as relative frequencies and percentages (%). The difference between the groups was explored using Mann-Whitney and Kruskal-Wallis for continuous variables and Chi square for categorical variables. Significant variables were carried forward to regression analysis to identify the odds ratio (OR) of the variables independently associated with outcomes. OR were reported with 95% confidence interval (95% CI). Multivariate analysis was carried out to adjust crude OR for confounding factors.

All tests were two-sided and a p value = 0.05 was considered significant. All statistical analysis was performed using SPSS^©^ (version 23.0; SPSS Inc. Chicago, IL).

### 2.3 Ethics

This study was retrospective and included only fully-anonymised data from investigations and assessment performed as standard of care. As such, no ethical approval was required as confirmed by the Joint Compliance Office at Imperial College London.

## 3. Results

### 3.1 Study population

A total of 561 adult patients were admitted at Imperial College Healthcare NHS Trust with a diagnosis of COVID-19 up to the date of data collection. Overall, 193 patients had imaging of the liver dated within 1 year from the COVID-19 admission. Specifically, 61 (31%) had fat infiltration on imaging (CT scan or US), while 132 (66%) had no evidence of liver disease. Those with other causes of liver disease (n = 5, 3%) were excluded. Specifically, 4 patients were excluded as they reported excessive alcohol consumption, and 1 patient had autoimmune hepatitis: all 5 patients were discharged.

At the time of outcomes update collection (10^th^ June), 9(5%) patients were still in hospital, 125(65%) were discharged and 59 (30%) died. In those with a complete outcome (i.e. discharged from hospital or death), the median length of stay was 7 days (4–12). The cause of death was respiratory failure secondary to COVID-19 pneumonia in54 (91%) cases, respiratory failure secondary to pulmonary embolism in 2 (3%), stroke in 2 (3%) and acute kidney failure in 1 (2%) case. The rate of ICU admission was 19% (n = 38).

In the NAFLD group, median age was 60 (53–75) years, median BMI was 30.6 (27–33.8) kg and60% (n = 37) were male. As per medical records, only 20% (n = 13) of the NAFLD patients was already being followed-up by a liver service. At the time of the data collection, 1 (2%) patient was still in hospital, 42 (68%) were discharged and 18 (29%) did not survive. In those with a complete outcome, the median length of stay was 7 days (3–14). The overall rate of ICU admission was 18% (n = 11). The prevalence of comorbidities is displayed in **Table 1**, while the distribution deaths per age range in **Table 2**. Finally, the two groups were homogeneous in terms of comorbidities.

**Table 1:** Differences between the NAFLD cohort and the non NAFLD cohort among patients with COVID-19.

	NAFLD cohort	Non NAFLD cohort	P value*
	n = 61	n = 132	
	Median (IQR)	Median (IQR)	
**Age,** *years*	**60 (53–75)**	**70.5 (53–79)**	**0.046**
Missing cases = 0 (0%)			
**BMI,** *kg/m*^*2*^	**30.6 (27–33.8)**	**27.1 (23.3–30.9)**	**0.003**
Missing cases = 58 (30%)			
**Laboratory tests at presentation**			
**Hb,** *g/L*	133.5 (116–145)	128 (112–144)	0.06
Missing cases = 3 (1%)			
**PLT,** *10*^*9*^*/L*	186 (148–246)	196 (155–269)	0.2
Missing cases = 4 (2%)			
**Lymphocyte count,** *10*^*9*^*/L*	0.9 (0.7–1.3)	0.9 (0.6–1.3)	0.18
Missing cases = 3 (1%)			
**Creatinine,** *μmol/L*	89.5 (72–125)	101.5 (72–142)	0.35
Missing cases = 5 (2%)			
**Urea,** *mmol/L*	7.2 (4.9–9.7)	6.9 (4.2–12.2)	0.94
Missing cases = 5 (2%)			
**Total bilirubin,** *μmol/L*	11 (8–17)	11 (8–16)	0.63
Missing cases = 35 (18%)			
**ALT,** *IU/L*	31 (21–56)	24 (15–40)	0.06
Missing cases = 29 (15%)			
**ALP,** *IU/L*	93 (69–123)	81 (63–125)	0.35
Missing cases = 22 (12%)			
**Albumin,** *g/L*	31 (26–34)	30 (25–34)	0.35
Missing cases = 27 (14%)			
**Ferritin,** *μg/L*	838 (529–1781)	828 (391–1279)	0.39
Missing cases = 92 (47%)			
**CRP,** *mg/L*	**107 (49–184)**	**91.2 (55–181)**	**0.05**
Missing cases = 12 (6%)			
**D-dimer,** *ng/ml*	1384 (879–2086)	1559 (778–3008)	0.49
Missing cases = 107 (55%)			
**PT,** *sec*	14 (13.4–15.2)	14.1 (13.2–15.1)	0.35
Missing cases = 50 (26%)			
**Lactate,** *mmol/L*	1.3 (1–1.9)	1.3 (1–1.9)	0.89
Missing cases = 43 (22%)			
**Observations at presentation**			
**Systolic BP,** *mmHg*	123 (112–143)	133 (112–150)	0.45
Missing cases = 9 (2%)			
**Diastolic BP,** *mmHg*	75 (64–83)	75 (65–85)	0.62
Missing cases = 9 (2%)			
**HR,** *bpm*	90 (80–105)	90 (80–104)	0.31
Missing cases = 9 (2%)			
**RR,** *br/min*	20 (18–28)	21 (18–28)	0.68
Missing cases = 9 (2%)			
**TC,** *°C*	36.9 (36.5–38.1)	37.1 (36.6–37.8)	0.5
Missing cases = 9 (2%)			
**EWS**	4 (2–6)	4 (2–7)	0.84
Missing cases = 9 (2%)			
	**NAFLD cohort**	**Non NAFLD cohort**	P value*
	**N = 61**	**N = 132**	
	**N (%)**	**N (%)**	
**Comorbidities**			
**Male gender**	36 (60)	85 (64)	0.28
Missing cases = 0 (0%)			
**Type-2 diabetes**	29 (47)	47 (35)	0.07
Missing cases = 0 (0%)			
**Hypertension**	26 (42)	66 (50)	0.21
Missing cases = 0 (0%)			
**Dyslipidaemia**	14 (23)	32 (24)	0.49
Missing cases = 0 (0%)			
**Ischaemicheart disease**	12 (19)	18 (13)	0.06
Missing cases = 0 (0%)			
**Lung disease**	11 (18)	14 (11)	0.25
Missing cases = 0 (0%)			
**CKD**	8 (13)	17 (13)	0.51
Missing cases = 0 (0%)			

Body Mass Index (BMI), Haemoglobin (Hb), platelets (PLT), alanine aminotransferase (ALT), alkaline phosphate (ALP), C reactive protein (CRP), protrombin time (PT), partial thromboplastin time (aPTT), Blood pressure (BP), heart rate (HR), respiratory rate (RR), temperature (TC), Early weaning score (EWS), chronic kidney disease (CKD). *P value* for the difference.

**Table 2:** Differences in terms of CRS stratification and outcomes between the NAFLD cohort and the non NAFLD cohort among patients with COVID-19.

	NAFLD cohort	Non NAFLD cohort	P value*
	n = 61	n = 132	
	n (%)	n (%)	
**CRS category at presentation**			
not present	26 (43)	47 (35)	0.54
1	13 (21)	32 (23)	0.21
2	6 (10)	12 (9)	0.34
3	14 (23)	39 (28)	0.66
4	2 (3)	2 (1)	0.87
Not present vs 1–4	26 (43) vs 35 (57)	47 (35) vs 85 (65)	0.21
1 vs 2–4	13 (21) vs 22 (36)	32 (23) vs 53 (38)	0.55
1–2 vs 3–4	19 (31) vs 16 (26)	44 (32) vs 41 (29)	0.49
1–3 vs 4	33 (54) vs 2(3)	83 (60) vs 2 (1)	0.33
	**NAFLD cohort**	**Non NAFLD cohort**	P value*
	**n = 61**	**n = 132**	
	**Median (IQR)**	**Median (IQR)**	
**Outcomes**			
**Overall length of stay,** *days*	7 (3–14)	6.5 (4–11)	0.72
**ICU length of stay,** *days*	9 (7–13)	11 (7–15)	0.11
**Time from onset of symptoms to admission,** *days*	**5 (3–7)**	**7 (4–10)**	**0.035**
**Time from onset of symptoms to ICU,** *days*	13 (8–20)	14 (9–20)	0.79
**Time from onset of symptoms to outcome,** *days*	11 (2–26)	10 (2–16)	0.76
	**NAFLD cohort**	**Non NAFLD cohort**	P value*
	**n = 61**	**n = 132**	
	**n (%)**	**n (%)**	
**Admission to ICU**	11 (18)	27 (20)	0.42
**Overall in-hospital mortality**	18 (29)	41 (31)	0.4
**< 40 years**	1 (1)	0 (0)	0.12
**41–50 years**	1 (1)	3 (2)	0.43
**51–60 years**	6 (10)	4 (3)	0.12
**61–70 years**	3 (5)	1 (1)	0.39
**71–80 years**	5 (8)	14 (10)	0.46
**> 81 years**	2 (3)	19 (14)	0.051

Cytokine release syndrome (CRS), intensive care unit (ICU). *P value* for the difference between the two groups.

### 3.2 NAFLD vs non-NAFLD population

#### 3.2.1 Differences between NAFLD and non-NAFLD population

When the NAFLD cohort was compared to the non-NAFLD cohort, those with NAFLD were significantly younger (60 vs 70.5 years, p = 0.046) and had a higher BMI (30.6 vs 27.1 kg/m^2^, p = 0.003). At presentation, the NAFLD group had significantly higher CRP levels (107 vs 91.2 mg/L, p = 0.05) compared to the non NAFLD group. However, the distribution of patients across the CRS categories was not significantly different between the two groups. In terms of outcomes, there was no difference in rates of admission to ICU and in-hospital mortality. Notably, those with NAFLD tended to present to the hospital earlier than those without NAFLD (5 vs 7 days, p = 0.035) (**Table 2**).

In the study population, male gender (OR 2.4, 95%CI: 1.2–5.1, p = 0.013), age (OR 1.03, 95%CI: 1.01–1.06, p = 0.001), lymphocyte count (OR 0.4, 95% CI: 0.23–0.89, p = 0.02) and CRS category (OR 1.3, 95% CI: 1.08–1.7, p = 0.01) were predictive factors of in-hospital mortality (**S1 Table**). Of note, the presence of NAFLD was not associated with admission to ICU (OR1.14, 95% CI: 0.53–2.5, p = 0.69) or with in-hospital mortality (OR0.86, 95% CI: 0.44–1.69, p = 0.67), after adjusting for confounding factors (age, male gender, type-2 diabetes, hypertension and dyslipidaemia).

#### 3.2.2 Difference between NAFLD and non-NAFLD stratified for metabolic factors

The whole population was then stratified for the components of metabolic syndrome based on the presence of type-2 diabetes, hypertension and dyslipidaemia. The presence of overweight/obesity was not included in the analysis as BMI values were incomplete in 58 (30%) patients.

Overall, 23 (12%) patients had 3 metabolic risk factors. In this sub-analysis, there was no difference between NAFLD (n = 9, 4%) and non-NAFLD (n = 148) group in terms of clinical parameters and observations at admission. Also, there was no difference in terms of admission to ICU (p = 0.66) and in-hospital mortality (p = 0.37).

Sixty-one (31%) patients were clustered for the presence of 2 metabolic risk factors. There was no difference between NAFLD (n = 39, 19%) and non-NAFLD (n = 3612%) presence in terms of clinical parameters and observations. Moreover, there was no difference in terms of admission to ICU (p = 0.43) and in-hospital mortality (p = 0.16).

A total of 51 (26%) patients had only one metabolic risk factor. Again, there was no difference between NAFLD (n = 20, 10%) and non-NAFLD (n = 31, 16%) subgroup in terms of clinical observations, including admissions to ICU (p = 0.55) and in-hospital mortality (p = 0.43).

Finally, 58 (30%) patients did not present any metabolic risk factor. In this subgroup, there was no difference between NAFLD (n = 7) and non NAFLD (N = 51) patients in terms of clinical parameters, admission to ICU (p = 0.27) and in-hospital mortality (p = 0.07).

### 3.3 Factors associated with mortality in the NAFLD population

In the sub-group of patients with NAFLD, we analysed the difference between those who died (n = 18) and those who were discharged (n = 42) from the hospital (**Table 3**). The cause of death was respiratory failure secondary to COVID-19 pneumonia in 16 (89%) and stroke in 2 (11%) cases. One patient still hospitalised (1%) at the time of data collection was excluded from this sub-analysis of outcomes.

**Table 3:** Differences between patients who died and those discharged in the cohort of patients with NAFLD and COVID-19.

	NAFLD cohort, discharged	NAFLD cohort, deaths	P value*
	n = 42	n = 18	
	Median (IQR)	Median (IQR)	
**Age,** *years*	59 (51–78)	60.5(53–75)	0.77
Missing cases = 0 (0%)			
**BMI,** *kg/m*^*2*^	30 (26–34.1)	30.2(26.7–33.5)	0.84
Missing cases = 11 (18%)			
**Laboratory tests at presentation**			
**Hb,** *g/L*	137 (124–145)	138(114–161)	0.75
Missing cases = 0 (0%)			
**PLT,** *10*^*9*^*/L*	194 (151–297)	167(139–216)	0.053
Missing cases = 0 (0%)			
**Lymphocyte count,** *10*^*9*^*/L*	1 (0.7–1.7)	0.9(0.5–1.2)	0.13
Missing cases = 0 (0%)			
**Creatinine,** *μmol/L*	83 (70–114)	91(74–184)	0.47
Missing cases = 1 (1%)			
**Urea,** *mmol/L*	6.7 (4.7–9.4)	7.8(4.5–14.1)	0.88
Missing cases = 1 (1%)			
**Total bilirubin,** *μmol/L*	11 (7–17)	15.5(8–22.7)	0.14
Missing cases = 11 (18%)			
**ALT,** *IU/L*	30 (14–56)	31(24–57)	0.5
Missing cases = 6 (9%)			
**ALP,** *IU/L*	92 (64–120)	98 (64–144)	0.86
Missing cases = 6 (9%)			
**Albumin,** *g/L*	31 (27–36)	31(24–34)	0.55
Missing cases = 11 (18%)			
**Ferritin,** *μg/L*	**688 (393–1275)**	**2076(781–3147)**	**0.003**
Missing cases = 19 (30%)			
**CRP,** *mg/L*	57.5 (22–136)	73.9 (33.7–140)	0.88
Missing cases = 3 (5%)			
**D-dimer,** *ng/ml*	1177 (567–1880)	1175(890–2391)	0.51
Missing cases = 30 (49%)			
**PT,** *s*	**13 (12.9–14.8)**	**14.4(13.4–16.1)**	**0.04**
Missing cases = 13 (21%)			
**aPTT,** *s*	33 (30–36.2)	35.7 (31.2–41)	0.35
Missing cases = 9 (2%)			
**Fibrinogen,** *g/L*	5.81 (4.9–7.4)	5.74 (3.7–7.3)	0.87
Missing cases = 9 (2%)			
**Troponin,** *ng/L*	**10 (5–19)**	**34 (30–41)**	**0.02**
Missing cases = 22 (36%)			
**LDH,** *U/L*	**314 (251–451)**	**498 (338–691)**	**0.025**
Missing cases = 41 (67%)			
**Lactate,** *mmol/L*	**1.2 (1–1.5)**	**1.7 (1.32–2.2)**	**0.002**
Missing cases = 13 (21%)			
**BNP,** *ng/L*	11 (10–20.8)	34 (10–398)	0.51
Missing cases = 5 (8%)			
**Observations at presentation**			
**Systolic BP,** *mmHg*	124 (112–141)	130 (114–147)	0.4
Missing cases = 5 (8%)			
**Diastolic BP,** *mmHg*	75 (65–82)	82 (67–88)	0.2
Missing cases = 5 (8%)			
**HR,** *bpm*	90 (83–103)	92 (80–114)	0.97
Missing cases = 5 (8%)			
**RR,** *br/min*	**20 (18–24)**	**28 (19–36)**	**0.01**
Missing cases = 5 (8%)			
**TC,** *°C*	36.9 (36.2–37.9)	37.5 (36.6–38.6)	0.55
Missing cases = 5 (8%)			
**EWS**	**3 (2–5)**	**7 (3–9)**	**0.009**
Missing cases = 5 (8%)			
	**NAFLD cohort, discharged**	**NAFLD cohort, deaths**	P value*
	**n = 42**	**n = 18**	
	**n (%)**	**n (%)**	
**Male gender**	**21 (50)**	**10 (71)**	**0.04**
Missing cases = 0 (0%)			
**Type-2 diabetes**	17 (40)	8 (44)	0.5
Missing cases = 0 (0%)			
**Hypertension**	17 (40)	7 (38)	0.41
Missing cases = 0 (0%)			
**Heart disease**	6 (16)	3 (21)	0.63
Missing cases = 0 (0%)			
**Lung disease**	8 (25)	2 (14)	0.69
Missing cases = 0 (0%)			
**CKD**	3 (9)	2 (14)	0.31
Missing cases = 0 (0%)			
**FIB-4 > 1.45**	19 (45)	7 (38)	0.38
Missing cases = 23 (37%)			
**FIB-4> 3.25**	16 (38)	7 (38)	0.61
Missing cases = 23 (37%)			
**Liver cirrhosis**	3 (7)	3 (16)	0.24
Missing cases = 0 (0%)			

Body Mass Index (BMI), Haemoglobin (Hb), platelets (PLT), alanine aminotransferase (ALT), alkaline phosphate (ALP), C reactive protein (CRP), protrombin time (PT), partial thromboplastin time (aPTT), blood pressure (BP), heart rate (HR), respiratory rate (RR), temperature (TC), Early weaning score (EWS), chronic kidney disease (CKD), Fibrosis index-4 (FIB-4). *P value* for the difference between the two groups.

More male patients died in the NAFLD group (71% vs 50%, p = 0.04). Also deceased patients in the NAFLD group had significantly higher levels of ferritin (2076 vs 688 μg/L, p = 0.003), PT (14.4 vs 13 s, p = 0.04), LDH (498 vs 314 U/L, p = 0.025), lactate (1.7 vs 1.2 mmol/L, p = 0.002) and troponin (34 vs 10 ng/L, p = 0.02). Also, those who died presented with significantly higher RR (28 vs 20 br/min, p = 0.01) and EWS (7 vs 3, p = 0.009) compared to those who survived (**Table 3**).

On multivariate analysis, only male gender (OR 2.7, 95% CI: 1.2–5.7, p = 0.01) ferritin levels (OR 1.002, 95% CI: 1.000–1.002, p = 0.043) and EWS (OR 1.1, 95% CI: 1.05–1.6, p = 0.049) were independently associated with in-hospital mortality, after adjusting for confounding factors (**Table 4**). Notably, ferritin (p = 0.06) and EWS (p = 0.21) were not associated with in-hospital mortality in the non-NAFLD group.

**Table 4:** Odd ratios for factors associated with in-hospital mortality in the NAFLD cohort.

		In-hospital mortality			
		Crude OR (95% CI)	*P value*	Adjusted OR (95% CI)*	*P value*
**Variable**	**Comparator vs reference**				
**Male gender**	**Male vs female**	**2.7 (1.3–5.6)**	**0.005**	**2.7 (1.2–5.7)**	0.01*
		**OR (95% CI)**	***P value***	Adjusted OR (95% CI)**	***P value***
**Ferritin**		**1.001 (1.00–1.02)**	**0.011**	**1.002 (1.000–1.002)**	**0.043**
**Lactate**		**4.8 (1.39–17)**	**0.013**	1.3 (0.98–1.7)	0.057
**LDH**		1.007 (1.00–1.01)	0.66	1.007 (0.99–1.01)	0.19
**Troponin**		1.002 (0.99–1.01)	0.79	1.004 (0.98–1.02)	0.64
**PT**		**1.86 (1.03–3.34)**	**0.038**	2.4 (0.86–6.7)	0.09
**RR**		**1.097 (1.017–1.18)**	**0.017**	1.02 (0.98–1.07)	0.1
**EWS**		**1.3 (1.07–1.66)**	**0.009**	**1.1 (1.05–1.6)**	**0.049**
		**OR (95% CI)**	***P value***	Adjusted OR (95% CI)***	***P value***
**FIB-4 > 1.45**	**Intermediate/high risk vs low risk**	1.5 (0.42–5.44)	0.42	1.02 (0.22–4.6)	0.97
**FIB-4 > 3.25**	**High risk vs Low/intermediate risk**	1.07 (0.31–3.6)	0.9	1.07 (0.15–3.5)	0.7
**Liver cirrhosis**	**Present vs absent**	2.4 (0.4–13.2)	0.32	1.47 (0.57–3.9)	0.48

Lactate dehydrogenase (LDH), protrombin time (PT), respiratory rate (RR), Early weaning score (EWS), Fibrosis index-4 (FIB-4).

* P-value for regression analysis adjusted for age, presence of type-2 diabetes, hypertension, dyslipidaemia.

** *P-value* for regression analysis adjusted for age, male gender, presence of type-2 diabetes, hypertension, dyslipidaemia.

***P-value for regression analysis adjusted for male gender, presence of type-2 diabetes, hypertension, dyslipidaemia

### 3.4 Severity of liver disease and outcomes

In the NAFLD group, 6 (9%) patients had a diagnosis of cirrhosis: 3 with Child-Pugh A, 2 with Child-Pugh B and 1 with Child-Pugh C. Fibrosis index-4 (FIB-4) was available in 38 (62%) patients (calculated on blood tests dated within 1 year but before the admission for COVID-19). According to FIB-4 values, the NAFLD group was stratified into 3 categories: 20 (33%) with low-riskFIB-4 (FIB-4> 1.45), 7 (11%) with intermediate-risk FIB-4 (1.45 <FIB-4 > 3.25) and 11 (18%) with high-risk FIB-4 (FIB-4> 3.25). The difference between the groups is shown in Table 4. At the time of admission, the three groups divided by FIB-4 were significantly different in age at presentation (54 vs 60 vs 67 years, p = 0.019) and BNP values (10 vs 406 vs 603 ng/L, p = 0.001) (**Table 5**).

**Table 5:** Differences between patients stratified for categories of FIB-4 in the cohort of patients with NAFLD and COVID-19.

	Pts with low-risk FIB-4	Pts with intermediate-risk FIB-4	Pts with high-risk FIB-4	P value*
	n = 20	n = 7	n = 11	
	Median (IQR)	Median (IQR)	Median (IQR)	
**Age,** *years*	**54 (45–60)**	**60 (60–65)**	**67 (57–78)**	**0.019**
**BMI,** *kg/m*^*2*^	30.4 (27–33.4)	32.5 (31.4–33.8)	27.3 (25.2–30.6)	0.17
**Laboratory tests at presentation**				
**Hb,** *g/L*	136 (122–151)	120 (109–134)	134 (119–143)	0.49
**PLT,** *10*^*9*^*/L*	212 (1671–297)	185 (169–205)	133 (113–201)	0.051
**Lymphocyte count,** *10*^*9*^*/L*	0.5 (0.7–1.4)	0.9 (0.7–1.1)	1 (0.7–1.2)	0.93
**Creatinine,** *μmol/L*	84 (71–109)	106 (93–120)	113 (93–183)	0.14
**Urea,** *mmol/L*	5.6 (4.2–8.8)	9.8 (9.6–10.9)	8.4 (4.9–9.3)	0.06
**Total bilirubin,** *μmol/L*	10 (7–14)	9 (10–15)	19 (13–29)	0.11
**ALT,** *IU/L*	44 (29–75)	26 (16–28)	33 (30–57)	0.073
**ALP,** *IU/L*	98 (81–125)	55 (45–94)	119 (87–200)	0.052
**Albumin,** *g/L*	32 (27–34)	25 (24–29)	29 (23–33)	0.23
**Ferritin,** *μg/L*	1405 (509–2386)	744 (610–1737)	1146 (529–1781)	0.66
**CRP,** *mg/L*	85.7 (31.2–135.5)	182 (107–255.1)	52.2 (21.3–78.4)	0.19
**D-dimer,** *ng/ml*	1553 (948–2200)	2102 (874–2000)	1486 (1287–6054)	0.79
**PT,** *s*	13.9 (13.2–14.6)	14.3 (13.6–14.8)	14 (13.7–15.9)	0.61
**aPTT,** *s*	32.9 (29.6–34.6)	34.2 (32.3–35.6)	36 (32.3–42.8)	0.4
**Fibrinogen,** *g/L*	6.04 (4.96–8.46)	5.9 (4.9–7.6)	4.53 (3.4–5.2)	0.08
**Troponin,** *ng/L*	10 (5–24)	41 (30.5–50.7)	13.5 (8.2–21)	0.27
**BNP,** *ng/L*	**10 (10–19.7)**	**406 (10–817)**	**603 (496–710)**	**0.001**
**Lactate,** *mmol/L*	1.1 (1–1.35)	1.5 (1.35–1.7)	1.3 (1.1–1.45)	0.37
**Observations at presentation**				
**Systolic BP,** *mmHg*	120 (111–135)	112 (102–116)	122 (118–131)	0.38
**Diastolic BP,** *mmHg*	75 (66–82)	69 (67–87)	70 (60–77)	0.68
**HR,** *bpm*	88 (78–110)	91 (88–93)	93 (82–100)	0.87
**RR,** *br/min*	19 (18–25)	18 (16–36)	24 (20–30)	0.28
**TC,** *°C*	37.39 (36.5–38.4)	36.7 (36.5–37)	36.9 (36.5–37.6)	0.87
**EWS**	5 (2–6)	3 (2–6)	5 (4–6)	0.85

Fibrosis index-4 (FIB-4), Body Mass Index (BMI), Haemoglobin (Hb), platelets (PLT), alanine aminotransferase (ALT), alkaline phosphate (ALP), C reactive protein (CRP), protrombin time (PT), partial thromboplastin time (aPTT), chronic kidney disease (CKD). Blood pressure (BP), heart rate (HR), respiratory rate (RR), temperature (TC), Early weaning score (EWS). *P value* for the difference between the three groups.

On multivariate analysis, the presence of FIB-4> 1.45 (OR 1.02, 95% CI: 0.22–4.6, p = 0.97), the presence of FIB-4 >3.25 (OR 1.07, 95% CI: 0.15–3.5, p = 0.7) or the presence of established cirrhosis (OR 1.47, 95% CI: 0.57–3.9, p = 0.48) were not significantly associated with in-hospital mortality (**Table 5**). Similarly, they were not associated with ICU admission.

## 4. Discussion

The outbreak of COVID-19 has become a public health emergency worldwide [21]. The increasing number of cases has led to unprecedented efforts at containment, given the rapid spread in the community, the high mortality among critically ill patients and the lack of treatment. So far, studies have suggested that older age, the presence of comorbidities and the development of acute respiratory distress syndrome are associated with increased mortality [22]. As such, there is much interest in phenotyping those who may be at risk for severe COVID-19 in order to develop specific surveillance or containment measures.

NAFLD represents an increasing cause of liver disease and is expected to become the leading cause of liver transplantation worldwide, as a consequence of the epidemic of metabolic risk factors [8]. Overall, NAFLD is associated with increased morbidity and mortality from both liver and non-liver related events (i.e cardiovascular disease (CVD)) with fibrosis stage representing the main prognostic factor [11]. As patients with NAFLD carry a particular combination of comorbidities (hypertension, diabetes, obesity and CVD), it has been argued that this group may also be at high risk for severe COVID-19 infection [15].

In this cohort of 193 patients with liver imaging available, admitted for COVID-19 in central London, we found that the overall prevalence of NAFLD was 30%. When compared to the non-NAFLD cohort, there was no difference in terms of overall mortality or age-stratified mortality (**Table 2**). Similarly, there was no difference in terms of ICU admission rates. However, given the strong age-dependent effect on outcomes in COVID-19, it is worthy to mention that patients with NAFLD were significantly younger than those without NAFLD (60 vs 70.5 years, p = 0.046) (**Table 1**). In terms of prognostic factors, the presence of NAFLD *per se* was not associated with adverse outcomes in the whole study population.

Increasing evidence suggests that COVID-19 infection can be bi-phasic [23]. An early phase is directly related to the virus pathogenic effect (flu-like phase) and may be followed by a relatively late phase (cytokine release syndrome (CRS)-like phase) in a subset of patients [6]. The cytokine storm appears to be the main reason for clinical deterioration and high mortality [4]. In this study, we found that the NAFLD group showed higher CRP (107 vs 91.2 mg/L, p = 0.05) levels compared to the non-NAFLD, although they were distributed equally among CRS categories (**Table 2**). These results suggest a more pronounced inflammatory status at presentation in patients with NAFLD. Interestingly, these results were unlikely due to a delayed admission, as those with NAFLD also tended to present to the hospital earlier since the onset of symptoms (5 vs 7 days, p = 0.035).

Another interesting finding was that the inflammatory status did not differ when the whole population was stratified for other metabolic risk factors. Notably, given the high numbers of missing BMI values, the presence of overweight/obesity was not included in this sub-analysis. As such, it might be argued whether obesity could have a major impact on CRP values, as those with NAFLD were heavier than those without NAFLD (BMI 30.6 vs 27.1, p = 0.003). In line with this, recent evidence has suggested that higher BMI is associated with adverse outcomes in COVID-19 [24]. Further studies are required to assess the impact of body weight among patients with NAFLD and COVID-19 infection.

An enhanced inflammatory response was particularly evident among the NAFLD patients who died during the admission. In particular, the NAFLD patients who died were more frequently men and presented with higher inflammatory markers (ferritin, PT and LDH) compared to NAFLD patients who survived. Moreover, the NAFLD patients who died had higher troponin levels (34 vs 10 ng/L, p = 0.034) in the context of normal BNP, suggesting the presence of a lower cardiac reserve in response to stress. Furthermore, the Early Weaning score (EWS), an algorithm which is usually applied for triaging patients at risk for acute deterioration [19], was also significantly higher (7 vs 3, p = 0.001) at presentation among those with NAFLD who did not survive. Of note, there was no difference in terms of liver function tests between the two groups, suggesting that liver injury was not a discriminant factor in this population. On regression analysis, ferritin and EWS at presentation were independently associated with mortality in the NAFLD group, highlighting the importance of the inflammatory storm on outcomes. Of note, male gender was also independently associated with mortality in the NAFLD group, confirming a well-known gender-based difference in terms of clinical outcomes from this infection [25]. Further studies will need to explore the role of gender, ferritin and EWS as predictors of outcome in NAFLD patients hospitalised with COVID-19.

When the NAFLD cohort was stratified according to FIB-4 risk categories, only age and BNP increased significantly with FIB-4 (Table 5). Moreover, the presence of intermediate/high risk FIB-4 as well as the presence of liver cirrhosis were not associated with adverse outcomes in the NAFLD cohort. These results suggest that the severity of COVID-19 in patients with NAFLD is not attributable to the severity of underlying liver disease, but rather other factors, which could include the host inflammatory response in view of the correlation with surrogates of inflammation. Another important finding of this study was that only 20% of patients with NAFLD admitted for COVID-19 were followed-up by a liver service, emphasizing the undiagnosed cases of NAFLD in the general population.

Our study presents some limitations. Firstly, the authors acknowledge that the study population was relatively small; however, we included consecutive patients with imaging of the liver available or known diagnosis of NAFLD, providing a more reliable selection of the study cohort: NAFLD vs non-NAFLD patients. Moreover, some of the data collected were incomplete, as shown in **Tables 1** and **3**. However, for the essence of this retrospective data collection, we relied on information recorded as part of the clinical assessment. Secondly, as the NAFLD patients were not followed-up in a specialist setting, results from other non-invasive markers of fibrosis (i.e. liver stiffness measurements) and/or liver histology scores were not available, reducing the accuracy in stratifying for severity of liver disease. Nevertheless, we opted for the calculation of FIB-4, currently recommended as a screening tool in the general population [26]. Thirdly, the number of patients with established NAFLD associated cirrhosis was probably insufficient to draw definitive conclusions. Finally, a follow-up and outcomes after discharge were not included in the analysis.

In conclusion, in this study, we report that the presence of NAFLD *per se* was not associated with adverse outcomes in our cohort of hospitalised COVID-19 positive patients, and the presence of advanced liver disease was not associated with adverse outcomes in the NAFLD population. Nevertheless, NAFLD patients were significantly younger at presentation, although there was no difference in terms of age-stratified mortality. Mortality in the NAFLD group was associated with male gender and with a particularly-pronounced host inflammatory response, with ferritin and EWS at presentation as main predictors. Further studies are needed to ascertain the role of the host inflammatory response in influencing mortality in this group.

## Supporting information

S1 Table Odd ratios for factors associated with in-hospital mortality in the whole cohort. (DOCX)

## List of abbreviations

NAFLD: Non-alcoholic fatty liver disease
EWS: Early weaning score
FIB-4: Fibrosis-4 index
ICU: intensive care unit
CRP: C reactive protein
SARS-CoV-2: severe acute respiratory syndrome coronavirus-2 (SARS-CoV-2)
SARS-CoV: severe acute respiratory syndrome coronavirus
MERS-CoV: Middle East respiratory syndrome coronavirus
ARDS: acute respiratory distress syndrome
MOF: multi-organ failure
CRS: Cytokine-release syndrome
NASH: Non-alcoholic steatohepatitis
RT-CRP: real-time reverse transcriptase-polymerase chain reaction
HR: heart rate
RR: respiratory rate
BP: blood pressure
TC: temperature
IQR: interquartile range
OR: Odd ratio
95% CI: 95% confidence interval
BMI: body mass index
