# Supplementary material for: In-hospital mortality is associated with inflammatory response in NAFLD patients admitted for COVID-19
Source: PLoS One. 2020 Oct 8;15(10):e0240400. doi: 10.1371/journal.pone.0240400 (PMC7544083; doi:10.1371/journal.pone.0240400)
Supplement: S1 Table — (DOCX) [file pone.0240400.s001.docx]

**S1 Table. Odd ratios for factors associated with in-hospital mortality in the whole cohort.**

|  | | **In-hospital mortality** | | | |
| --- | --- | --- | --- | --- | --- |
|  |  | **Crude OR (95% CI)** | ***P value*** | **Adjusted OR (95% CI)*** | ***P value*** |
| **Variable** | **Comparator vs reference** |  |  |  |  |
| **Gender** | **Male vs female** | **2.7**  **(1.3-5.6)** | **0.005** | **2.4**  **(1.2-5.1)** | **0.013*** |
| **Age** |  | **1.04**  **(1.02-1.06)** | **0.0001** | **1.03**  **(1.01-1.06)** | **0.001*** |
| **Lymphocytes count,** *10^9^/L* |  | **0.44**  **(0.22-0.84)** | **0.0001** | **0.4**  **(0.23-0.89)** | **0.02*** |
| **CRS category** |  | **1.4**  **(1.1-1.8)** | **0.0001** | **1.3**  **(1.08-1.7)** | **0.01*** |

*CRS: cytokine release syndrome.*

** P-value* for regression analysis adjusted for presence of type-2 diabetes, hypertension, dyslipidaemia.
